# Canopy attachment position influences metabolism and peel constituency of European pear fruit

**DOI:** 10.1186/s12870-018-1544-6

**Published:** 2018-12-18

**Authors:** Sara Serra, Nathanael Sullivan, James P. Mattheis, Stefano Musacchi, David R. Rudell

**Affiliations:** 1 Tree Fruit Research and Extension Center, Washington State University, Wenatchee, WA 98801 USA; 2 Tree Fruit Research Laboratory, Agricultural Research Service, U.S. Department of Agriculture, Wenatchee, WA 98801 USA

**Keywords:** Postharvest, Storage, Quality, Ripening, Orchard environment, Light, Non-targeted metabolomics, Triterpene, Phenylpropanoid, Superficial scald, *Pyrus communis* L

## Abstract

**Background:**

Inconsistent pear fruit ripening resulting from variable harvest maturity within tree canopies can contribute to postharvest losses through senescence and spoilage that would otherwise be effectively managed using crop protectant and storage regimes. Because those inconsistencies are likely based on metabolic differences, non-targeted metabolic profiling peel of ‘d’Anjou’ pears harvested from the external or internal canopy was used to determine the breadth of difference and link metabolites with canopy position during long-term controlled atmosphere storage.

**Results:**

Differences were widespread, encompassing everything from expected distinctions in flavonol glycoside levels between peel of fruit from external and internal canopy positions to increased aroma volatile production and sucrose hydrolysis with ripening. Some of the most substantial differences were in levels of triterpene and phenolic peel cuticle components among which acyl esters of ursolic acid and fatty acyl esters of *p*-coumaryl alcohol were higher in the cuticle of fruit from external tree positions, and acyl esters of α-amyrin were elevated in peel of fruit from internal positions. Possibly the most substantial dissimilarities were those that were directly related to fruit quality. Phytosterol conjugates and sesquiterpenes related to elevated superficial scald risk were higher in pears from external positions which were to be potentially rendered unmarketable by superficial scald. Other metabolites associated with fruit aroma and flavor became more prevalent in external fruit peel as ripening progressed and, likewise, with differential soluble solids and ethylene levels, suggesting the final product not only ripens differentially but the final fruit quality following ripening is actually different based on the tree position.

**Conclusions:**

Given the impact tree position appears to have on the most intrinsic aspects of ripening and quality, every supply chain management strategy would likely lead to diverse storage outcomes among fruit from most orchards, especially those with large canopies. Metabolites consistently associated with peel of fruit from a particular canopy position may provide targets for non-destructive pre-storage sorting used to reduce losses contributed by this inconsistency.

**Electronic supplementary material:**

The online version of this article (10.1186/s12870-018-1544-6) contains supplementary material, which is available to authorized users.

## Background

Inconsistent pear (*Pyrus communis* L.) fruit maturity at harvest has practical and economic consequences to pear producers, marketers, retailers, and, ultimately, consumers. Cold storage, typically at temperatures as low as − 0.5 °C, is necessary for many European pear cultivars, including ‘d’Anjou’, to both trigger ripening and, paradoxically, reduce the rate of ripening with the intent of delivering properly ripened, unblemished fruit to the retail shelf [1]. Because handling of even slightly ripened pears can lead to damage, producers often sort and package fruit prior to placing them into hypoxic controlled atmosphere (CA) cold storage (for ‘d’Anjou’, 1.5 kPa O_2_, > 1 kPa CO_2_), which is commonly used to extend “green life” [2]. Consequences can include inconsistent ripeness on the retail shelf, spoilage within packed boxes and, potentially, removing pears from the boxes following storage and repackaging them prior to shipping.

Tree position can influence on-tree maturity as well as many fruit ripening-related processes contributing to overall fruit quality of rosaceous tree fruit species, including apple and pear. This inconsistency is exaggerated on larger pear trees [3] yet is also manifest in smaller and more compact canopies [4]. Pear quality traits altered by tree position throughout storage can include both red blush and background color, soluble solids content, and titratable acidity [4]. Secondary metabolites comprising these traits are likewise impacted although, perhaps as it may be obvious with regard to metabolites directly linked with quality traits, little work has been reported related to metabolism during ripening as influenced by tree position. Pear [4] and apple [5] ethylene production and respiration following harvest are also influenced by tree position supporting that quality-related phenotypes were affected altogether by differential ripening and may actually result in an altered phenotype of pears received by the consumer.

A combination of factors may contribute to quality differences and underlying metabolism as influenced by canopy position. Fruit proximity to assimilate sources and other sinks as well as production and translocation of auxin, gibberillins, and cytokinins in remote organs outside the fruit during development may be contributing factors [6]. Along with ethylene, these plant growth regulators govern much of fruit development, maturation, and ripening [7]. Light environment also influences apple [8] and pear [3] maturity, quality, and postharvest behavior, even on opposite sides of the same fruit [9]. Orchard temperature, which may be expected to be different between external and more shaded portions of the tree as well as different sides of the fruit, can also substantially influence postharvest behavior [10]. Our preliminary survey of ‘d’Anjou’ metabolites without cold storage indicated that multiple pathways may be influenced by tree position including those likely related to light exposure such as flavonol glycosides, chlorophyll levels, and potentially quality-related metabolites including sugars [11]. This study was limited yet warranted more comprehensive long-term storage comparisons using non-targeted metabolic profiling to determine the scope of processes altered by tree position.

Non-targeted metabolic profiling is a group of one or more techniques using instrumentation, such as gas and liquid chromatography coupled with mass spectroscopy as well as mass spectroscopy or NMR alone to evaluate metabolites from multiple pathways from a single sample [12]. Such techniques attempt to limit bias and approach comprehension with varying degrees of success depending upon the sample evaluated. Non-targeted metabolic profiling has been employed to establish relationships among factors that challenge apple and pear fruit during harvest and cold chain transitions [13]. To date, much of these efforts have been directed towards identifying pathways and metabolic interplay involved in apple and pear postharvest disorder genesis and development including high CO_2_ stress of pear [14] and apple [15], firm flesh browning of apple [16], soft scald and soggy breakdown of apple [17, 18], and superficial scald of apple [19–21]. Additionally, similar approaches targeting metabolites from multiple pathways have been applied to determine peel metabolism of green and red ‘d’Anjou’ [22], flesh metabolism of ‘La France’ during fruit development and following a 1 month room temperature postharvest period [23], and in seeds and different tissues of ‘Radana’ pears [24].

Global metabolic analyses generate large datasets that may confound univariate tests. Instead, multivariate tests, such as principal components analysis (PCA), are often used to determine if major experimental factors impacted the metabolome and, even, which of the metabolites were associated with each factor [25]. However, these analyses principally focus on major, often known, sources of variance such as experimental contrasts or only variables comprising most of the variance in a dataset and are not suitable for describing highly dimensional data [26]. Correlation network analyses are particularly suited to discovering less obvious, yet biologically significant, functions within a time-series experiment revealing areas of metabolic co-regulation as influenced by other factors incorporated in the experiment [27]. Network models can be generated using pairwise correlation tables and closely associated regions highlighted using a variety of network topological techniques [27].

In this study, we sought to determine how peel metabolism may be altered during long term CA storage depending upon whether fruit was harvested from internal and external regions of the large canopy ‘d’Anjou’ trees. We expected that metabolic differences would be attributable to key components of peel appearance as well as ripening rate and quality.

## Results

**Fig. 1:**
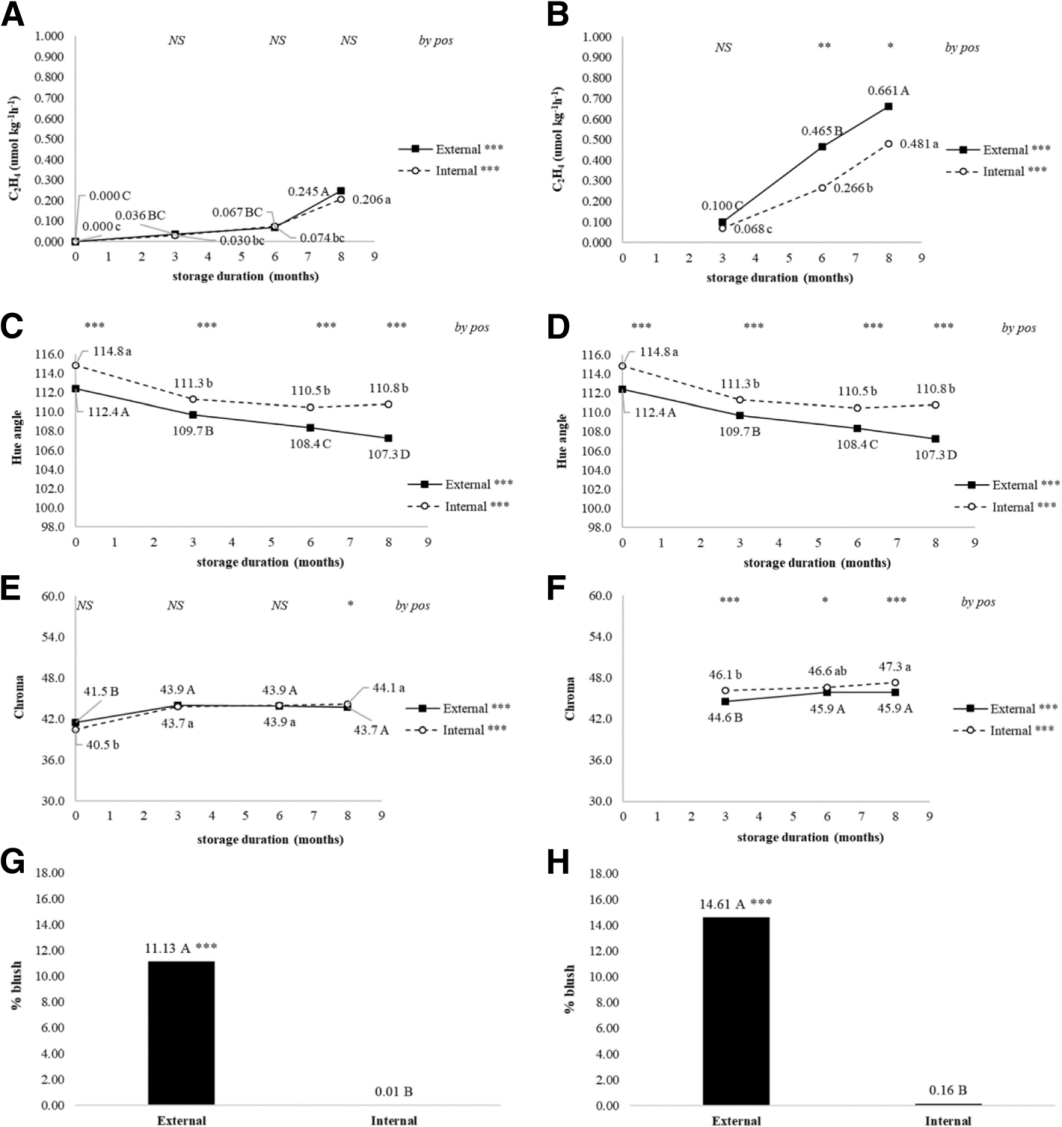
Ethylene evolution (**a**, **b**), hue angle (**c**, **d**), chroma (**e**, **f**), and percent blush. Changes were recorded during 8 months of controlled atmosphere storage of ‘d’Anjou’ pears harvested from different tree positions. Additional fruit were ripened at 23 °C for 7 days following each sampling from storage (**b**, **d**, **f**, **h**) Significance was tested using proc. GLM in SAS, type III sums of squares. Means were compared using a post-hoc Bonferroni test. Means followed by the same letter are not different at *p* < 0.05. Significance of overall change during storage is indicated by asterisks (NS, not significant; *, *p* < 0.05; **, *p* < 0.01; ***, *p* < 0.001) on the right side of the legend labels. Letters indicate significant differences among storage durations (months) for fruit harvested from the external (uppercase) or internal (lowercase) canopy. At the top of each sampling date asterisks indicate significance of difference between external and internal canopy for each storage duration (NS, not significant; *, *p* < 0.05; **, *p* < 0.01; ***, *p* < 0.001)

**Fig. 2:**
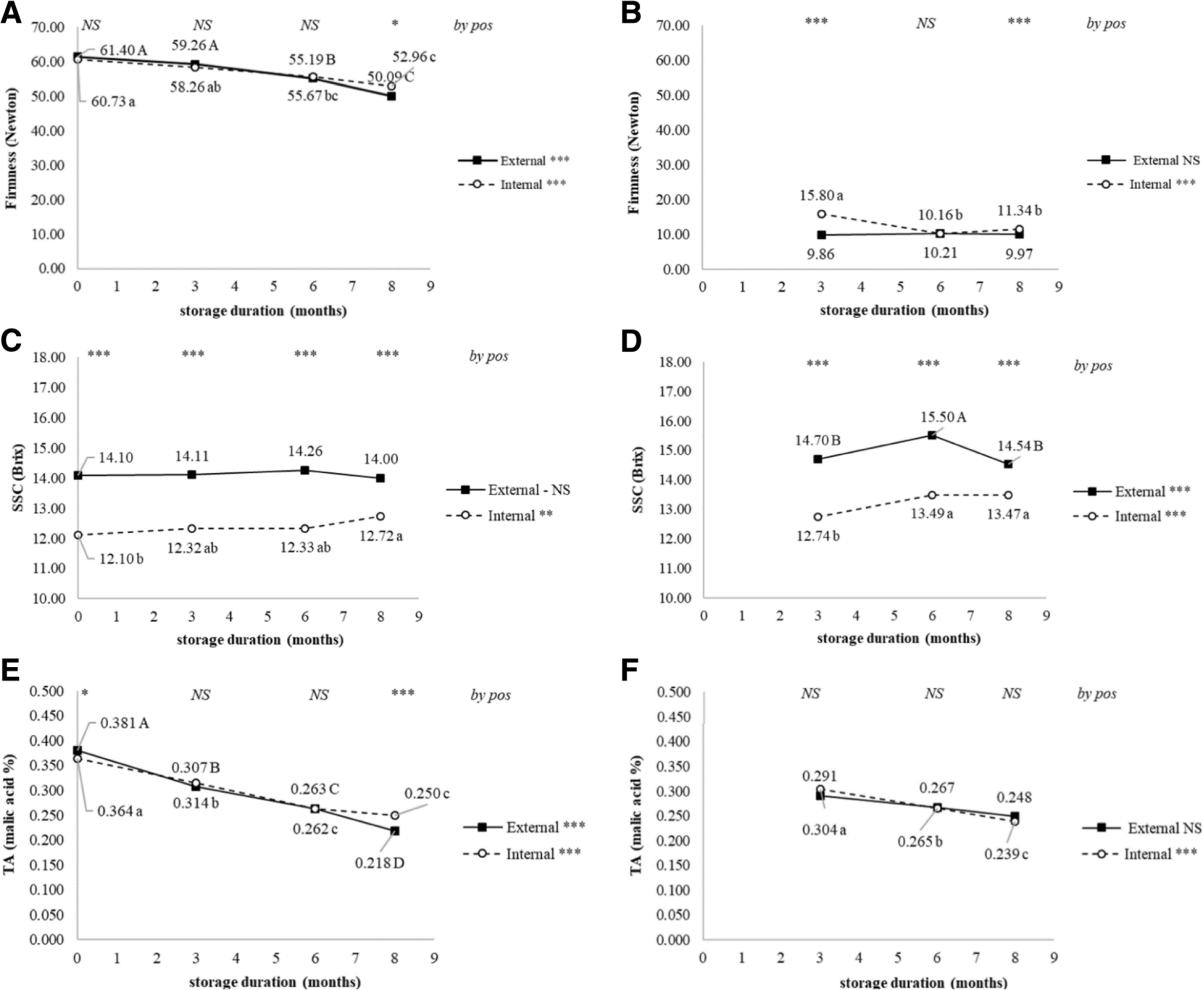
Fruit firmness (**a**, **b**), soluble solids (**c**, **d**), and titratable acidity (**e**, **f**). Changes were recorded during 8 months of controlled atmosphere storage of ‘d’Anjou’ pears harvested from different tree positions. Additional fruit were ripened at 23 °C for 7 days following each sampling from storage (**b**, **d**, **f**). Significance was tested using proc. GLM in SAS, type III sums of squares. Means were compared using a post-hoc Bonferroni test. Means followed by the same letter are not different at *p* < 0.05. Significance of overall change during storage is indicated by asterisks (NS, not significant; *, *p* < 0.05; **, *p* < 0.01; ***, *p* < 0.001) on the right side of the legend labels. Letters indicate significant differences among storage durations (months) for fruit harvested from the external (uppercase) or internal (lowercase) canopy. At the top of each sampling date asterisks indicate significance of difference between external and internal canopy for each storage duration (NS, not significant; *, *p* < 0.05; **, *p* < 0.01; ***, *p* < 0.001)

Standard indicators of ripening and quality were influenced by tree position at one or more time points both during storage and after a 7 d ripening period following each storage duration. Ethylene production immediately after removal from storage was no different after 8 months but was greater by external fruit following the 7 d ripening period after 6 and 8 months confirming that ripening occurred first in the external fruit (Fig. 1 a and b). Internal fruit remained firmer during storage and following the 7 d ripening period at 3 and 8 months (Fig. 2 a and b). Soluble solids content was elevated in external cortex at harvest, during storage, and following the 7 d ripening periods indicating higher sugar content in cortex of external fruit although differences do not indicate any association with ripening (Fig. 2 c and d). Titratable acidity was only lower in external fruit after 8 M without ripening period (Fig. 2 e and f).

**Fig. 3:**
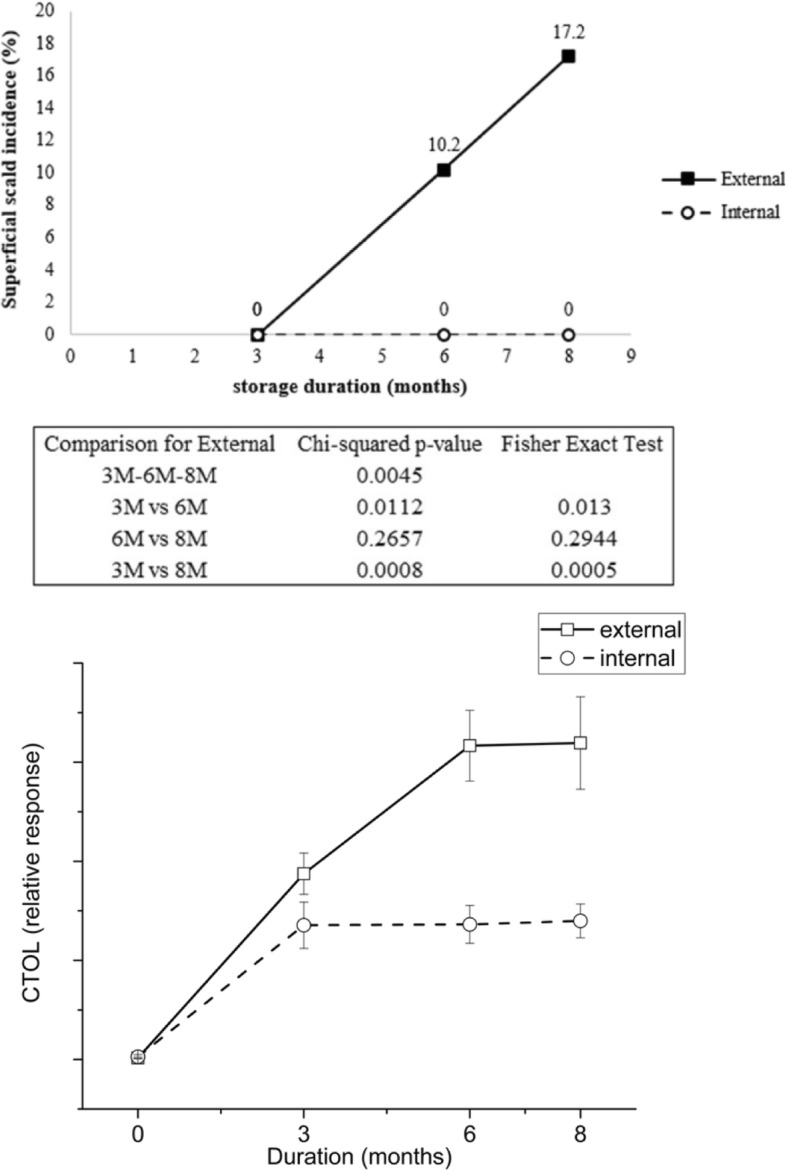
Superficial scald symptoms (**a**) and (7*E*,9*E*)-2,6,10-Trimethyl-2,7,9,11-dodecatetraen-6-ol (CTOL) levels (**b**). Only external fruit developed superficial scald. For Fig. 5a, scald incidence is indicated above the symbols and significance of time point comparisons are indicated both by Chi-squared *p*-value and Fisher’s Exact test. For Fig. 5b, error bars indicate standard error (*n* = 5). Where error bars are not visible, standard error was smaller than the symbol size

Peel appearance was impacted by tree position at harvest, during storage, and following the 7 d ripening period in a manner consistent with pear fruit ripening. Percent total blush was substantially higher on external fruit (Fig. 1 g and h). Hue angle (°h) of internal peel was higher throughout the study with external peel transitioning from green to yellow during the 7 d post-storage ripening periods (Fig. 1 c and d). Likewise, chroma, which indicates color saturation, was elevated in internal peel throughout the study (Fig. 1 e and f). Superficial scald developed only on external fruit following the 7 d ripening period after 6 and 8 M storage (Fig. 3).

### Metabolic profile

Characteristic changes of quality-related traits were manifest in changes of related metabolites although metabolism, as influenced by tree position and storage duration, was far more widespread than that obviously linked with fruit quality. Evaluated metabolites were all freely solubilized by the extraction systems employed, therefore polymers were not evaluated. In total, 816 metabolites were detected with 187 identified or tentatively identified (Additional file 1: Table S1). Many compounds or even compound classes appear to be novel to pear fruit literature including glycocerebrosides, amyrin acyl esters, cycloartenol acyl esters, uvaol and erythrodiol acyl esters, and ursanoic/oleanolic esters. When possible, identity of metabolites was established by comparison with standards or those synthesized in-house, while identification of others relied on mass spectral interpretation, comparison with standards with similar mass spectra, and/or comparison with published mass spectra. The level of identification of each metabolite detected in the study is indicated in Additional file 1: Table S1 with additional information regarding identification of metabolites focused on in the current report (Additional file 2: Table S2).

### Principal components analysis

**Fig. 4:**
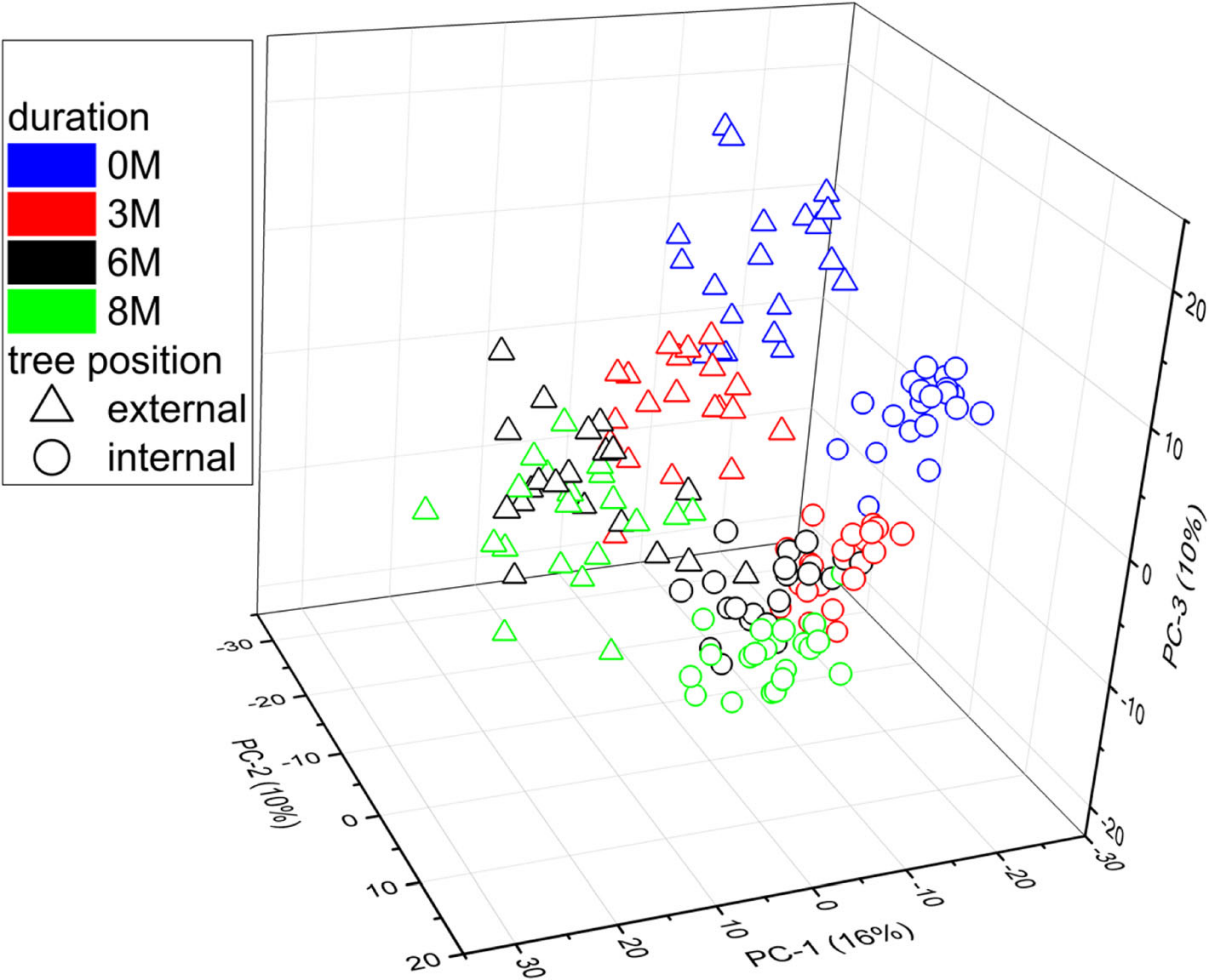
Principal components analysis (PCA) scores plots of the ‘d’Anjou’ pear peel metabolome. Differences revealed include those based on tree position and changes with storage duration. Each score represents 816 metabolites and indicate differences between fruit peel throughout storage as well as changes in the metabolome during storage

Principal components analysis indicated the major sources of variance were contributed by storage duration and tree position (PC1, 16%; PC2, 10%; PC3, 10%). The model accounted for relatively little variance indicating the high dimensionality of these data and the possibility of other more latent relationships among metabolites. The low total variance accounted for also may indicate the model is not suitable for linking metabolites with either of the main experimental factors (Fig. 4). PCA scores of internal and external fruit were nearly entirely separated over the whole storage duration in these dimensions while those from external fruit changed more over time than those of internal fruit where 3–8 months scores were similar. This not only confirms the impact tree position has on metabolism at harvest but that it also continues to change differentially during storage.

### Metabolic network

**Fig. 5:**
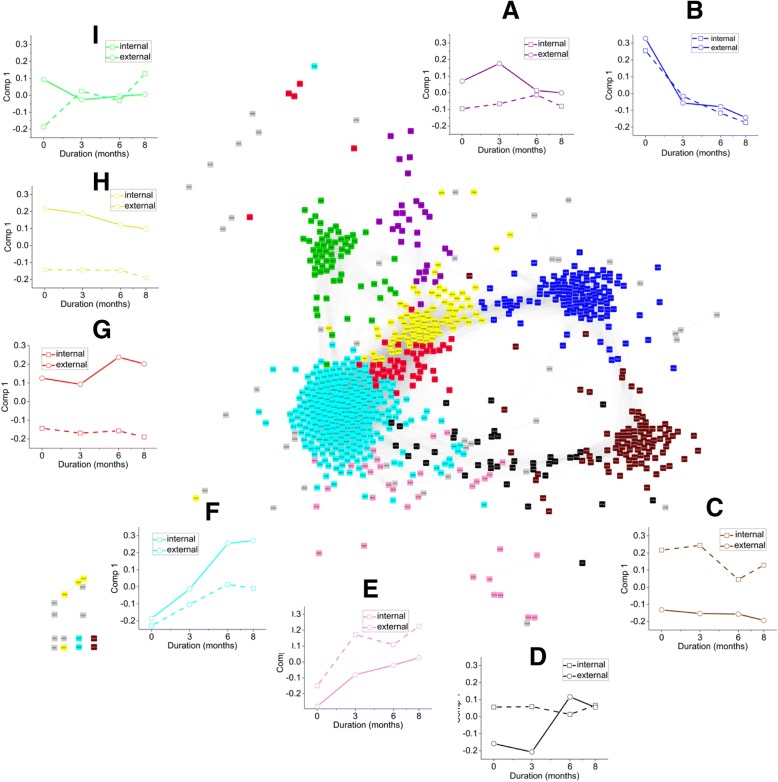
Weighted gene correlation network analysis (WGCNA) signed metabolic network of ‘d’Anjou’ pear peel. Pears were harvested from different tree positions and stored in controlled atmosphere for up to 8 months. Nodes of the same color indicate metabolites reside within the same module. Co-proximity of edges indicate closeness and (inter)connectivity of edges with neighbors and further indicates relationships among modules. Eigen(metabolites)genes summarize member metabolite levels within each module over storage of each tree position (**a**-**i**). The network analysis characterizes minor functions within this highly dimensional data set

A metabolic network generated from all metabolites (nodes) and connected by edges produced 10 modules, 7 of which formed well-defined “neighborhoods” following the layout process (Fig. 5). Examination of the WGCNA eigengene, or “eigenmetabolite” in this case, reveals levels of metabolites (blue module) higher at the beginning of storage that decrease during storage in peel of both internal and external fruit (Fig. 5 b). These compounds including xanthophylls and 6 carbon volatile aldehydes are associated with relatively less ripe fruit. Levels of metabolites in the turquoise module increased during storage albeit more so in peel of external fruit (Fig. 5 f). These two modules illustrate how the given spatial arrangement of modules within the network reflects changes over storage time for internal and external peel. Examination of module positions within the network additionally indicate that the brown module contains metabolites with higher levels in internal fruit which are relatively stable over time while yellow and red modules are the opposite with respect to fruit position. These relationships are confirmed by changes predicted by each module eigenmetabolites (Fig. 5 c, h, g). The positions of the green and purple modules are less clear with respect to storage duration or tree position (Fig. 5 a and i). The pattern in the green module potentially indicates coordinated metabolism, although there was no clear association with key experimental inputs other than higher levels in external peel at harvest.

### Metabolism represented in each module

The blue module is associated with compounds most abundant in unripe fruit that diminish with storage duration and, likewise, ripeness with little difference between tree positions (Fig. 5 b). Many compounds typically more prevalent in unripe fruit were found in this module. This includes 6 carbon volatile aldehydes and esters, amino acids, and organic acids typically associated with green fruit as well as ß-carotene and xanthophylls. A variety of triglycerides also reside in this module.

Conversely, the turquoise module contains those compounds that increase with storage duration although more so in external peel. The eigenmetabolite ultimately levels off by 6 months and indicates concentrations generally increased to greater final levels during storage in external fruit. This module contains a variety of metabolites expected to increase with ripening including alcohols, esters, and other aroma components linked with pear ripening (Fig. 5 f and Additional file 1: Table S1). Also, other processes typically occurring during storage such as sucrose (blue module) hydrolysis producing fructose and glucose (turquoise module) also support this relationship with ripening as do increasing levels of leucine and isoleucine. Amino acids and primary metabolites that increased with ripening included succinic acid and galactaric acid. Chlorogenic acid (3-caffeoylquinic acid) was present in this module. The majority of acyl esters of *p*-coumaryl alcohol were also present in the turquoise module.

**Fig. 6:**
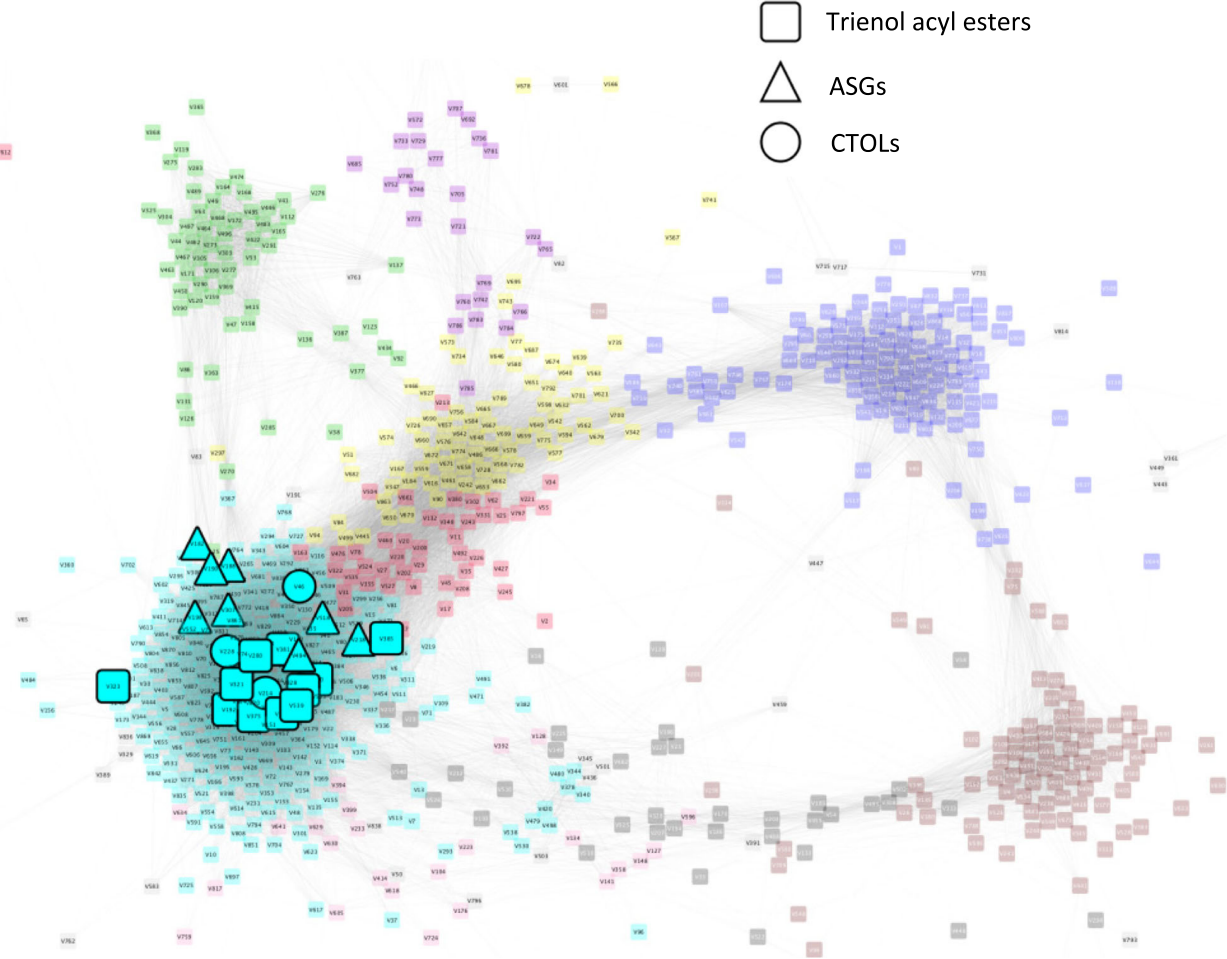
Weighted gene correlation network analysis (WGCNA) signed metabolic network of ‘d’Anjou’ pear peel. Pears were harvested from different tree positions and stored in controlled atmosphere for up to 8 months. Metabolites associated with scald risk are highlighted within the network. Highlighted metabolites include acylated steryl glycosides (ASGs; triangle), conjugated trienol (CTs; circles), and trienol acyl esters (squares) (Table 1; Additional file 2: Table S2) within the WGCNA network. Increasing levels of these classes of compounds during storage has been linked with superficial scald risk of apple fruit and was the case in pear peel in the current study

Compounds residing in this module represent other pathways not previously linked with ripening or other phenotypes such as superficial scald risk (Fig. 6; Table 1). Following the 23 °C post-storage ripening period, peel of external fruit was less green and developed more superficial scald (Figs. 1 and 3). Among these are multiple partially identified cerebrosides, acylated steryl glycosides (ASGs), conjugated trienols (CTOLs), fatty acyl esters of primary and secondary farnesols, and acyl esters of *p*-coumaryl alcohol. The turquoise module also contains a number of isoprenoids linked with oxidative stress and superficial scald risk of apple and pear. (7*E*,9*E*)-2,6,10-Trimethyl-2,7,9,11-dodecatetraen-6-ol (CTOL) and another related trienol as well as a variety of farnesyl acyl conjugates increased with ripening mostly in the external peel. This relationship was not limited to the sesquiterpenoids as the module also contains many acylated steryl glycosides (ASGs). Other triterpenoids included sitosteryl and campesteryl linoleate and pentacyclic triterpenol and triterpenoic alcohol acyl conjugates, including erythrodiol and uvaol esters, and 2 ursanoic acyl esters.

**Table 1:** Superficial scald risk associated metabolites highlighted in Fig. 6

Node label	Identification	module	symbol
V68	*Trienol linoleate* ^*a*^	turquoise	square
V147	*unknown trienol acyl ester*	turquoise	square
V160	*unknown trienol acyl ester*	turquoise	square
V192	Farnesyl stearate	turquoise	square
V280	Farnesyl linoleate^a^	turquoise	square
V321	Farnesyl linolenate	turquoise	square
V375	*Trienol oleate*	turquoise	square
V381	Farnesyl oleate	turquoise	square
V539	*unknown farnesol acyl ester*	turquoise	square
V385	*unknown sequiterpenoid*	turquoise	square
V323	*unknown sequiterpenoid*	turquoise	square
V214	(7E,9E)-2,6,10-Trimethyl-2,7,9,11-dodecatetraen-6-ol^a^	turquoise	circle
V228	*unknown trienol*	turquoise	circle
V46	*unknown trienol*	turquoise	circle
V188	campesteryl glucosyl linoleate	turquoise	triangle
V190	ß-sitosteryl glucosyl linoleate	turquoise	triangle
V307	stigmasteryl glucosyl linoleate	turquoise	triangle
V494	stigmasteryl glucosyl linolenate	turquoise	triangle
V518	stigmasteryl glucosyl palmitate	turquoise	triangle
V218	*Unknown stigmasteryl fatty acyl ester*	turquoise	triangle
V182	ß-sitosteryl glucosyl linolenate	turquoise	triangle
V198	ß-sitosteryl glucosyl palmitate	turquoise	triangle

^a^See Additional file 2: Table S2 for information supporting identification

List includes compounds tentatively identified using mass spectral interpretation and/or comparison or those classified as a certain class of compound (italicized). The module label, color, and symbol are also indicated

**Fig. 7:**
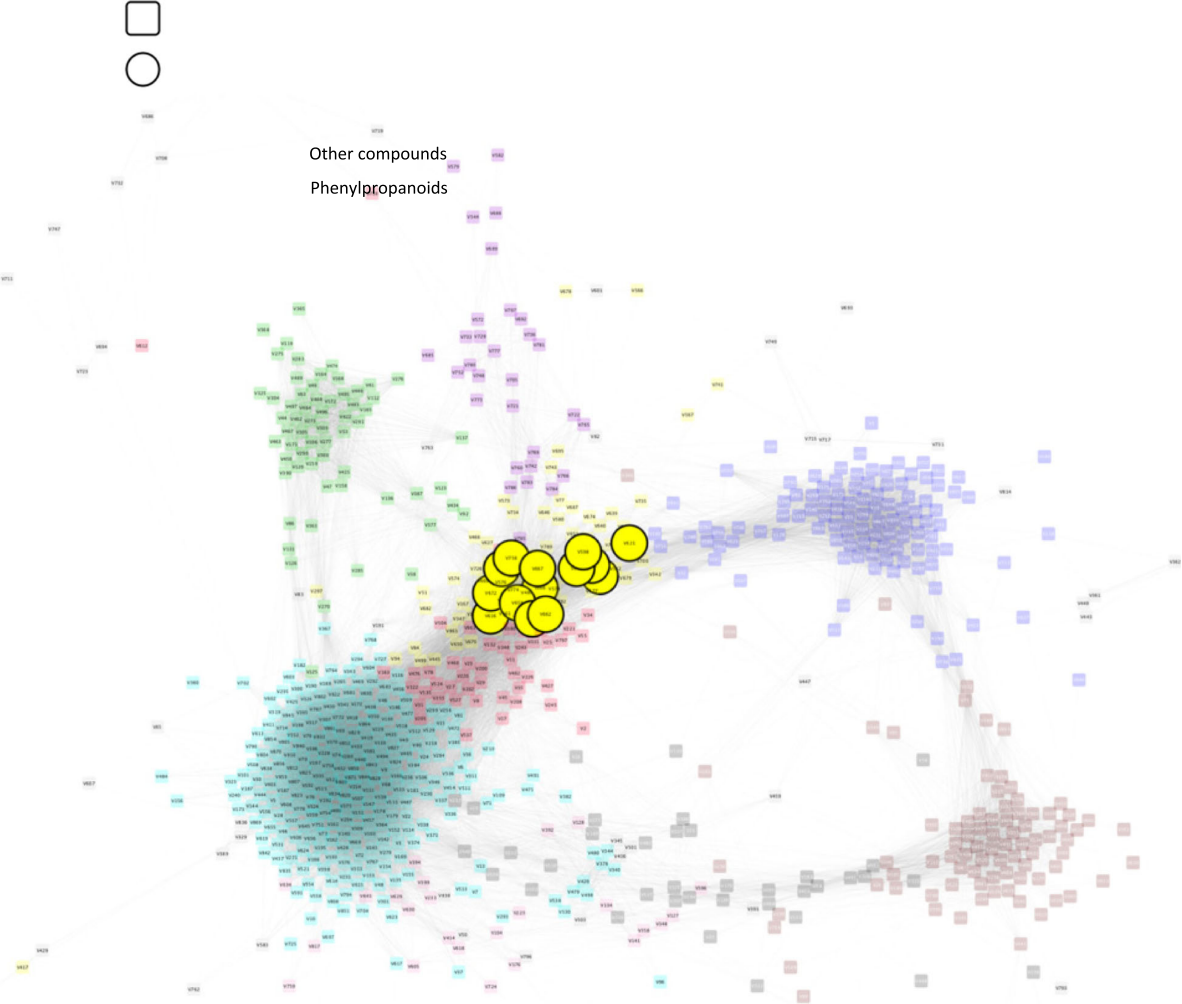
Weighted gene correlation network analysis (WGCNA) signed metabolic network of ‘d’Anjou’ pear peel. Pears were harvested from different tree positions and stored in controlled atmosphere for up to 8 months. Highlighted compounds include identified and tentatively identified flavonol glycosides, hydroxy- and methoxycinnamic acids and conjugates, and arbutin (Table 2; Additional file 2: Table S2). Metabolites residing in the yellow module were found in higher concentrations in peel of ‘d’Anjou’ pears grown on external than internal positions of the tree canopy

Both the yellow and red modules are represented by compounds with elevated levels in external peel at harvest that change very little during storage, although the red module eigenmetabolite does indicate metabolites in external peel may increase (Fig. 5 g). Many phenolic compounds included in the yellow module are typically associated with greater light exposure such as flavonol glycosides as well as upstream metabolites or metabolites from different branches of the phenolic pathway (Fig. 7; Table 2). Metabolites in this module included hyperin, rutin, phloridzin, arbutin, caffeic acid, and ferulic acid. Other tentatively identified and partially characterized metabolites in this pathway included isorhamnetin rutinoside, isorhamnetin 3-O-(6′-acetyl)glucoside, quercetin 7-(6′-acetyl)glucoside, an unidentified caffeoylquinic acid, dicaffeoylquinic acids, and feruloylmalic acid (Additional file 2: Table S2). Fatty acyl esters of *p*-coumaryl alcohol are also highly represented in this module. Anthocyanin levels were not evaluated although they are reflected in the % blush evaluation.

**Table 2:** Phenylpropanoid metabolites highlighted in Fig. 7

Node label	Identification	module	symbol
V621	Phloridzin	yellow	circle
V756	*unknown chlorogenic acid* ^a^	yellow	circle
V649	*Dicaffeoylquinic acid* ^a^	yellow	circle
V589	Caffeic acid	yellow	circle
V594	Arbutin	yellow	circle
V542	Ferulic acid	yellow	circle
V672	*unknown hydroxycinnamic acid* ^a^	yellow	circle
V667	*Feruloylmalic acid* ^a^	yellow	circle
V666	Quercetin 7-(6″-acetylglucoside)^a^	yellow	circle
V662	Isorhamnetin 3-O-rhamnoglucoside^a^	yellow	circle
V658	Rutin	yellow	circle
V657	*Dicaffeoylquinic acid*	yellow	circle
V653	Isorhamnetin 3-(6″-acetylglucoside)^a^	yellow	circle
V616	Hyperoside	yellow	circle

^a^See Additional file 2: Table S2 for information supporting identification

List includes compounds tentatively identified using mass spectral interpretation and/or comparison or those classified as a certain class of compound (italicized). The module label, color, and symbol are also indicated

**Fig. 8:**
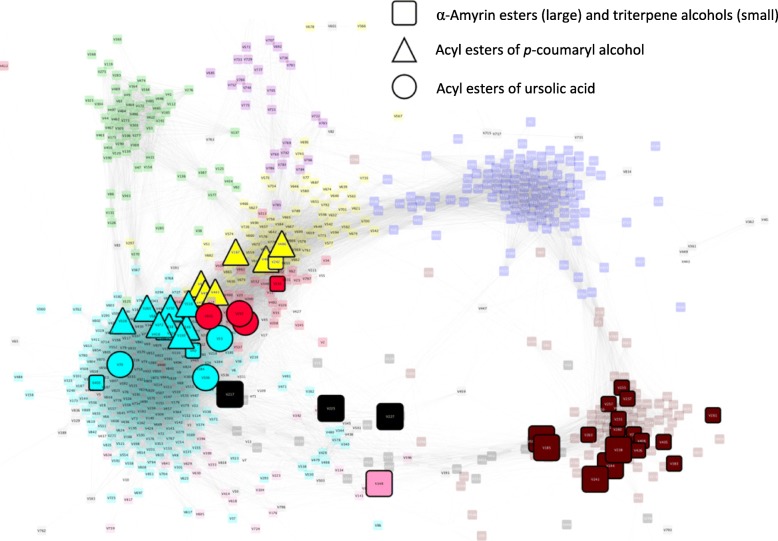
Weighted gene correlation network analysis (WGCNA) signed metabolic network of ‘d’Anjou’ pear peel. Pears were harvested from different tree positions and stored in controlled atmosphere for up to 8 months. Novel cuticular metabolites of interest (Table 3; Additional file 2: Table S2) are highlighted within the network. Large and small emboldened square nodes represent select α-amyrin acyl esters and unknown compounds classified as hydroxylated pentacyclic triterpenoic acids, respectively. Circles represent acyl esters of ursolic acid. Triangles represent fatty acyl esters of *p*-coumaryl alcohol

Trends in the red module are very similar to the yellow module, yet the module contains few relatively polar compounds. Triterpene acyl esters, putative lipids and other nonpolar metabolites primarily reside in this module (Fig. 5 g). Those identified and tentatively identified include pentacyclic triterpenol esters, phytosteryl esters and cycloartenyl esters as well as betulinic acid. The ursolic/oleanolic acid peak was saturated beyond the linear range of the HPLC column rendering accurate quantitation impossible with the current dataset. The red and turquoise modules contain all of the acyl esters of ursolic acid (Fig. 8; Table 3) as well as metabolites also comprised of the same principal [M + H]^+^ mass spectral peak as betulinic, ursolic, and oleanolic acids. These compounds have longer retention times than the triterpene moieties alone and have molecular ions [M-H]^−^ indicating they are acyl esters (C18:0, C20:0, and C22:0).

**Table 3:** Triterpenoid membrane and cuticular metabolites highlighted in Fig. 7

Module label	Identification	module	symbol
V185	3β,19α-dihydroxy-urs-12-en-28- oic acid (pomolic acid)^a^	brown	large square
V238	3β-?-p-coumaroyloxy-hydroxy-urs-12-en-28-oic acid	brown	large square
V244	3β-cis-p-coumaroyloxy-2α-hydroxy-urs-12-en-28-oic acid^a^	brown	large square
V396	3β-p-coumaroyloxy-dihydroxy-urs-12-en-28-oic acid	brown	large square
V148	3-oxo-1,19α-dihydroxy-urs-12-en- 28-oic acid (annurcoic acid)^a^	pink	large square
V241	3β-trans-p-coumaroyloxy-2α-hydroxy-urs-12-en-28-oic acid	brown	large square
V217	α-amyrin linolenate	black	large square
V225	α-amyrin linoleate	black	large square
V227	α-amyrin myristate	black	large square
V40	*unknown pentacyclic triterpenol*	turquoise	small square
V235	*ursolic aldehyde*	brown	small square
V237	*unknown pentacyclic triterpenol*	brown	small square
V251	*unknown pentacyclic triterpenol*	brown	small square
V253	*unknown pentacyclic triterpenol*	brown	small square
V257	*unknown pentacyclic triterpenol*	brown	small square
V260	*unknown pentacyclic triterpenol*	brown	small square
V263	*unknown pentacyclic triterpenol*	brown	small square
V405	*unknown pentacyclic triterpenol*	brown	small square
V406	*unknown pentacyclic triterpenol*	brown	small square
V426	*unknown pentacyclic triterpenol*	brown	small square
V242	*unknown pentacyclic triterpenol*	yellow	small square
V243	*unknown pentacyclic triterpenol*	red	small square
V403	*unknown pentacyclic triterpenol*	turquoise	small square
V383	*unknown pentacyclic triterpenol*	brown	small square
V261	*unknown pentacyclic triterpenol*	brown	small square
V116	*p*-coumaryl stearate^a^	turquoise	triangle
V130	*p*-coumaryl eicosanoate	turquoise	triangle
V146	*p*-coumaryl docosanoate	turquoise	triangle
V265	*p*-coumaryl oleate	turquoise	triangle
V272	*p*-coumaryl linoleate/*p*-coumaryl palmitate mix	turquoise	triangle
V292	*coumaryl heinicosanoate*	turquoise	triangle
V326	*p*-coumaryl linolenate	turquoise	triangle
V438	*coumaryl ester*	turquoise	triangle
V445	*coumaryl eicosanoate*	yellow	triangle
V167	*p*-coumaryl heinicosanoate	yellow	triangle
V461	*coumaryl ester*	yellow	triangle
V486	*coumaryl ester*	yellow	triangle
V499	*coumaryl ester*	yellow	triangle
V84	*coumaryl linoleate*	yellow	triangle
V202	Ursolyl eicosanoate	red	circle
V535	Ursolyl stearate	red	circle
V70	Ursolyl linolenate^a^	turquoise	circle
V506	Ursolyl oleate	turquoise	circle
V8	Ursolyl docosanoate	red	circle
V15	Ursolyl heinecosanoate	turquoise	circle

^a^See Additional file 2: Table S2 for information supporting identification

List includes compounds tentatively identified using mass spectral interpretation and/or comparison or those classified as a certain class of compound (italicized). The module label, color, and symbol are also indicated

Opposite of the yellow and red modules, metabolites found in the brown module (Fig. 5 c) do not change appreciably with storage duration but remain higher in peel from internal fruit. This module contains photosystem pigments including both chlorophyll a and b, lutein, and 2 compounds with similar spectra and retention times as lutein. In comparison with the yellow and red modules, the brown module contains more polar hydroxytriterpenes, dihydroxytriterpenes, and dihydroxytriterpenoic acids (Fig. 8; Table 2). These include multiple compounds tentatively identified as dihydroxylated triterpenoids (not including uvaol or erythrodiol), dihydroxytriterpenoic acids such as pomolic acid, annurcoic acid, and hydroxycinnamoyl ursanoic and dihydroxytriterpenoic conjugates (Additional file 1: Table S1).

The green and purple modules are less descriptive in relation to known experimental factors aside from metabolite levels generally starting higher in the external than internal peel and remaining at a similar level for the remainder of the storage period (Fig. 5 a and i). One interesting aspect is the similar, unique properties of compounds residing in these modules that, along with their close correlation, indicating these modules likely represent pathways or similarly regulated regions of pathways. Glucocerbrosides identified using mass spectra and authentic standards are included in the green module which, in fact, contains mostly odd masses and mass spectra similar to the glucocerebroside standards (Additional file 1: Table S1). Other members include monogalactosyldiacylglycerols (MGDGs), digalactosyldiacylglycerols (DGDGs), steryl glucosides (SGs), and other unidentified lipids. The purple module contains only compounds detected using the polar method also with odd isotopic masses. Most of the compounds in the purple module have not been identified.

The layout of the remaining modules (black, pink, and grey) were relatively spatially dispersed compared with the other modules possibly indicating metabolism within these modules is not as closely related. This may also indicate there is more variability in trends among metabolites within the same module. Generally, black and pink modules contain elevated levels of metabolites in internal peel at harvest and rising in both external and internal, in the case of the black, or only external, in the case of the pink module (Fig. 5 d and f). Of note, within the black module (Fig. 8; Table 2) are a number of α-amyrin acyl esters and other unidentified compounds containing amyrin-like mass spectrum and similar retention times (Additional file 2: Table S2). The grey module contains unassociated metabolites.

In summary, tree position did impact peel appearance throughout storage with external peel degreening more following 8 months plus 7 d as well as a higher incidence of superficial scald. Reflecting the visual phenotype, results not only indicate that the peel metabolic profile is different but also changes differentially during storage depending upon tree position during fruit development. These differences were manifested in a variety of pathways and, consequently it is difficult to summarize these changes with respect to common themes. Overall, phenolic levels were elevated in the external peel as were levels of acyl esters of *p*-coumaryl alcohol and many metabolites related to ripening processes. Other compounds increasing more in external peel were those typically associated with superficial scald risk, including CTOL, ASGs, methanol, and methyl esters. External peel did develop scald after 8 months CA storage plus 7 d ripening time. In general, sesquiterpenoid and triterpenoid metabolism seemed to differentiate beyond this as with evidence suggesting overall acylation of many pentacyclic triterpenoic alcohols and acylated steryl glycosides levels increasing more in external peel as storage progressed. In peel from internal fruit, acylated α-amyrin and free pentacyclic triterpenols and triterpenoic alcohols were more prevalent. Triglyceride levels were higher at harvest and decreased equally during and after storage in peel from both tree positions.

## Discussion

It is evident from prior reports that tree position can affect pear fruit harvest maturity and quality [3, 4]. However, the overall impact on metabolism reveals more bases for many of these differences. Expected changes of levels of metabolites with known associations with ripening confirmed the validity of experimentation and analysis providing confidence in links discovered between less understood metabolism and ripening, tree position, and peel appearance. These not only include those metabolites linked with photoprotection but also cuticle and cuticular wax modification and superficial scald risk typically associated with storage conditions. Finally, while metabolism is altered across storage, does tree position contribute to variability in the final product on the retail shelf given a degree of ripening?

### Assessment of the metabolome

Our survey of the metabolome is comprised of both previously reported as well as novel metabolites new to pear literature. Basic sugar, organic acid, aroma, and phenolic profiles are well established for European pear (*P. communis*) [28–30], especially with relationship to organoleptic quality [31]. Pear flesh polar metabolites have also been evaluated in a more global context including mono-, di-, and tri-saccharides, organic acids, and amino acids, many of which are included in the current report [14, 22]. Oikawa et al. [23], expanded their assessment of pear flesh to include additional polar secondary metabolites as well as lipids and phytohormones. Li et al. [22] also evaluated pear fruit phenolics along with primary metabolism. Many compounds in the current study remain unidentified, partially characterized, or tentatively identified based on mass spectral similarity with known compound or published mass spectra. We chose to focus any identification on compounds of interest with respect to tree position.

An earlier report [11] presents the scope of our profiling strategy which employs zwitterionic hydrophilic interaction chromatography to estimate not only sugars, organic acid, and amino acids but, also, relatively polar, non-volatile metabolites, including providing a more thorough assessment of phenylpropanoid pathway products particularly more prominent in peel than cortex [32]. Our analysis detects and has been used to identify, with varying degrees of confidence, an array of compounds included in earlier reports including quercetin and isorhamnetin glycosides, hydroxy and methoxy cinnamic acid esters, arbutin, and flavan-3-ols (Additional files 1 and 2: Tables S1 and S2; [28, 33, 34]).

European pear aroma quality and production during ripening and storage is relatively well described and has also been the subject of numerous articles [35–37]. The volatile profile reported here reflects existing information and includes aldehydes, alcohols, ketones, sesquiterpenes, and acyl esters expected during ripening [35–37]. Compounds such as ethyl and methyl decadienoate along with other esters and alcohols are largely responsible for “ripe” pear flavor profile [37, 38], and “grassy” C6 aldehydes prominent in the “unripe” profile of apple fruit aroma [39] were included in this evaluation.

This analysis also accounts for a number of non-polar metabolites of which there are few or no previous reports. The solvent system used with APCI-MS affords ionization of compounds of a range of polarities from polyhydroxylated pentacyclic triterpenoic acids to phytosterol conjugates. Chlorophyll and other non-polar pigments were recovered as may be expected from photosynthetically active tissue. CTOL [40] and acylated esters of farnesol [41] and CTOL acyl esters [18, 21] detected entirely in apple cuticle [42] were also found here. While not typically associated with pome fruit, apple peel and flesh also contain a compliment of different triglycerides [18, 43], 2-hydroxyacyl glucocerebrosides [43–45], and galactolipids [43, 46]. Acyl esters of *p*-coumaryl alcohol found in apple peel [47–49] were also detected in the current analysis in pear peel. Stigmasterol, ß-sitosterol, and campesterol (free sterols; FSs) as well as glycosides (SGs), acyl esters (SEs), and acylated steryl glycosides (ASGs) found in apples [46, 50, 51] were also detected. Identification of nonpolar metabolites was not complete, for instance, phosphatidylinositol diglyceride, phosphatidylethanolamine diglyceride, and other phospholipids present in apple are not reported here.

Pear peel contains a similar complex mixture of isomers, conjugates, and precursors of ursolic acid as associated apple cuticle and wax [52]. Using our analytical system, ursolic acid, which co-elutes with a smaller oleanolic acid peak, was the most prominent peak in the total ion chromatogram but the recovery exceeded the linear range of the system and, as a result, could not be used in our multivariate and network models. Ursolic, oleanolic, and betulinic acid were recovered from pear peel in earlier studies [24]. Other precursors and polyhydroxylated pentacyclic and esterified (hydroxycinnamate) ursanoic and oleanolic compounds including *p*-coumaryl and caffeoyl esters of ursolic acid [53–55], pomolic and annurcoic acid [54, 55], α- and ß-amyrin, lupeol, and uvaol [56] have been reported in apple peel and were also identified or tentatively identified here in pear (Additional files 1 and 2: Tables S1 and S2).

This list of pear fruit metabolites has been expanded with the identification of erythrodiol and acyl esters of α-amyrin, cycloartenol, uvaol, erythrodiol, and ursolic acid in the current report, all of which can be detected in apple [42]. The position of esterification of the uvaol and erythrodiol was not determined and the majority of this class of compounds remains to be identified as this is far from a comprehensive list. However, these compounds can be loosely classified given the retention time and mass spectral characteristics of identified compounds in this class. Peaks exhibiting mass spectra characteristic of pentacyclic triterpenoic alcohols and other triterpenol appear to reside within approximately the first 18 min (retention of α -amyrin) of the HPLC solvent program and beyond this retention time when esterified with fatty acids (Additional file 1: Table S1). To date, characterization of pentacyclic triterpene esters has been primarily performed on plant materials from Asteraceae [57]. Acyl esters of amyrin and ursolic acid were recovered from dewaxed epidermis and wax, respectively, of fruit and leaves from a number of species [58, 59], including apple peel [42]. While continued identification of new wax components highlights the complexity of the pear epidermis, it is also evident that our evaluation is far from comprehensive with respect to this system and any findings must be considered with that caveat.

### Validation using benchmark metabolic changes

One approach supporting the validity of methods used to assess the metabolome is to look for expected changes given a set of experimental conditions. Metabolism known to be related to ripening processes is probably the most demonstrable change that can be used for validation. For instance, multiple alcohols and esters responsible for ‘d’Anjou’ [36] and other European pear [38] ripe aroma, including methyl 2,4-decadienoate [37, 60], increased with storage duration establishing the relationship between the turquoise module and ripening during storage. Sucrose hydrolysis is a benchmark process of apple fruit metabolism during air [61, 62] and CA [16] storage. As may be expected given this association, sucrose resided in the blue module and diminished during storage while products of sucrose hydrolysis, glucose and fructose, were in the antithetical turquoise module supporting a relationship between relatively unripe fruit peel and compounds in the blue module.

### Relationships with light exposure

Relationships between compound levels and relative light exposure during fruit development are exhibited by metabolites either residing in modules related to external (yellow, red, and turquoise) or internal (brown, black) fruit. Average irradiance within the tree canopies that were the bases for assigning tree position have been previously published [3]. Chlorophyll a and b are both included in the brown module which, also, best reflects relative levels and changes in hue angle indicating chlorophyll was more prevalent in internal fruit and did not degrade during long-term CA storage. It is not entirely clear why peel shaded by the canopy would contain more chlorophyll as bagged Asian pears [63] contain reduced chlorophyll levels. It is possible that color differences merely reflect differences of maturity although this is not entirely obvious at harvest given that only soluble solid content and titratable acidity were different between internal and external fruit. Apple peel subjected to damaging levels of light also contain reduced chlorophyll levels [64, 65].

Red blush and levels of flavonol glycosides including hyperin, rutin, isorhamnetin 3-O-rhamnoglucoside, and isorhamnetin 3-O-(6′-acetylglucoside) were elevated in peel from external fruit (yellow module) may be more indicative of light exposure. Quercetin glycoside levels are elevated in Asian pear [63, 66] and apple peel [67, 68] following light exposure as well as relatively higher light based on canopy position [69]. Other phenolic compounds are also elevated in external peel including a 2 chlorogenic acids, hydroxycinnamic acids as well as arbutin and its precursor, ferulic acid. Chlorogenic acid (3-caffeoylquinate) resided in the turquoise module indicating a potential role of both light and ripening in the regulation of its biosynthesis. Chlorogenic acids are also elevated in apple peel exposed to high light leading to sunburn injury [70] or artificial UV light following harvest [68], although not always with respect to sun facing side compared to the opposite side [67] or internal tree position [69]. As in the current work, neither monomeric nor polymeric flavon-3-ol levels were influenced by light in apple peel [67, 68, 70].

While percent red blush and flavonol glycoside levels are some of the most evident indicators of sun exposure, other classes of epicuticular components also seem to have similar relationships with this condition. Fatty acyl esters of *p*-coumaryl alcohol levels are elevated in external peel as are acyl esters of pentacyclic triterpenoic alcohols, triterpenols, and triterpen-diols (Fig. 6; Table 1; Additional file 1: Table S1). Conversely, levels of many similar un-acylated triterpenes such as pomolic acid or annurcoic acid or hydroxycinnamoyl esters of triterpenoic alcohols were more prevalent in internal peel (brown module). Not all of the acyl triterpenol esters have been identified in the black module but those tentatively identified metabolites were α-amyrin esters while modules representing higher amounts in external peel contain the acyl esters of ursolic acid. Acyl esters of ursolic/oleanolic acid are primarily localized in ‘Granny Smith’ apple cuticle/wax zone while α-amyrin ester are extractable principally from the de-waxed peel [42] and the same appears to be true in the current study indicating α-amyrin esters, like phytosterol esters, are likely membrane rather than extracellular components such as ursolic acid and its acyl esters.

It is generally well understood that physical attributes of leaf epidermis are altered by different stressors including heat and light [71]. Apple cuticle and wax is thicker on more exposed portions of the fruit [72] and composition changes with ripening [73, 74]. Ursolic acid levels are also reduced on the exposed side of apple fruit [75] although the acyl esters of ursolic acid were elevated in peel from external pears. Other polyhydroxylated pentacyclic triterpenoic acids and hydroxycinnamic acid esters of these compounds were elevated in peel from internal fruit (brown module). It has been suggested that relatively low alkane and high triterpene levels may act to alter wax phase transition and cuticular permeability influencing gas exchange and water loss [76]. Fatty acyl esters of *p*-coumaryl alcohol content may be higher in pear peel receiving more light for different reasons. Acyl esters of hydroxycinnamic alcohols and other flavonols in the intercuticular zone may provide some measure of photoprotection by absorbing light within certain spectral bands [77]. Likewise, *p*-coumaryl alcohol esters in apple wax may serve an antioxidative function [48] although earlier work indicates they may merely be unpolymerized monomeric substrates for incorporation into cutin and suberin [47]. A recent report indicates that these alkyl esters of hydroxycinnamic acids are indeed free monomers of cutin whose *p*-coumaryl, caffeoyl, and feruoyl ratio and total amount, both free and polymerized, depends upon level of russet development and likely the environmental conditions leading to microcracking and russet development on apple [78]. Presence of elevated levels of unincorporated monomers may point to increased cuticular defects resulting from elevated irradiation in combination with ripening. Legay et al. [78] did not detect free alkyl hydroxycinnamic acids in nonrusseted peel as in pear in the current study.

### Tree position influences superficial scald incidence and levels of risk-related metabolites

The turquoise module may be associated with “ripe” metabolites but, possibly more relevantly, metabolites that increase more during storage in the external peel. From an appearance standpoint, the change with ripening was evident during storage given the relationship with color change (hue angle) typically associated with degreening [79], although this was not entirely supported by the chlorophyll levels. One important trait that was different between external and internal peel following 8 months CA plus 7 d at 20 °C was superficial scald development only on peel of external fruit (Fig. 5). Scald is a superficial necrotic peel disorder of apple and European pear [80] with symptoms that develop after long term storage. This was unexpected as scald purportedly develops on pears that are less mature at harvest [81] and less exposed to the sun [82], both conditions that were more linked with internal fruit in this study.

However, scald risk may have been predicted by elevated levels of a number of metabolites associated with apple and pear elevated scald risk that are members of the turquoise cluster (Fig. 6; Table 1). These include CTOL, a secondary alcohol produced by α-farnesene oxidation, associated with apple [40] and pear [83] scald risk. A volatile product of CTOL oxidation, 6-methyl-5-hepten-2-one (MHO) is associated with scald risk of apple [84] and was elevated in external pear peel (Fig. 5). Other metabolites associated with scald risk in apple included ASGs [51] as well as acylated secondary hydroxyfarnesene, methanol, and methyl acyl esters [21]. Regions of apple [20, 85] and ‘d’Anjou’ pear [81] peel receiving higher light prior to storage typically have reduced scald and CTOL levels. ‘D’Anjou pears are generally scald-free in the blushed (red) portion of external fruit peel indicating that the difference of scald development between external and internal was more related to factors other than overall light exposure. Regardless of the causes, which warrant further examination, tree position is clearly a risk factor and differences of tree position lead to entirely different metabolomes and can render fruit less or entirely unmarketable based on scald development.

### Differential ripening based on tree position

Hue angle, firmness, and ethylene production, all traits linked with pear ripening, were different following the 7 d ripening period after each storage pull out suggesting ripeness, and perhaps less obvious quality traits, were potentially impacted by tree position throughout the entire simulated supply chain. As pears finally reached a stage where they were too ripe to market, these data indicate that “final” fruit quality was altered and every management decision across the supply chain would be impacted resulting from inconsistency contributed by tree position. Furthermore, as superficial scald is associated with ripening, many other less obvious ripening-related processes are influenced to the point that the quality of fully ripe fruit may depend upon tree position. In fact, levels of many of the metabolites attributable to “ripe” pear quality continued to increase more in external fruit during storage as summarized by the turquoise module eigenmetabolite. Ester metabolites, especially methyl and ethyl decadienoate, are key components of ripe pear flavor as are many of the other alcohols and esters contained in this module [37]. Aroma is key to consumers’ perception of ripeness [86]. Similarly, glucose, fructose, and sorbitol levels were comparatively elevated in external peel potentially impacting sweetness. There are many other instances of metabolic disparity not related to appearance that may underlie differences of edible quality. As with those aforementioned, these differences may not only contribute to the overall inconsistency of the final product coming out of CA storage but also fruit quality on the retail shelf if post-storage fruit sorting is not employed.

## Conclusions

Pear ripening rate and final quality outcome is impacted by tree position even after common storage where ethylene and other volatile compounds may trigger or otherwise impact ripening. Quality variability is characterized by differences of appearance that become more pronounced as fruit ripened. Accordingly, metabolism underlying each of these appearance as well as other ripening related traits are altered by the position of the fruit in the canopy. This includes diverse pathways from those associated with apple and pear scald risk to flavor and aroma. Elevated levels of acylated ursolic acid and *p*-coumaryl alcohol were also apparent in the wax of external fruit although roles and complete pathway characterization remain undetermined. Comparison of the metabolic profile points to the role light may have in this disparity but also indicates the imposition of less evident factors given the unexpected relationship with scald and fruit receiving more light. Finally, a practical outcome of ripening differences associated with tree position can be costly post-storage sorting of riper fruit that can lead to scuffing and bruising or, worse yet, fruit loss. Horticultural practices reducing canopy size or pre-storage sorting using an accurate basis of contrast may provide more consistent stored product with more tailored storage and marketing strategies. Here, we have identified potential metabolite targets for sorting on the basis of relative tree position including those ostensibly associated with light exposure including flavonol glycosides or chlorophyll. It is possible that existing tools such as the differential absorbance (DA) meter, which is purportedly estimates primarily chlorophyll content below the peel, or other non-destructive optical devices may offer solutions for segregating pears according to tree position based on targeting these optically active components.

## Methods

### Fruit source, postharvest maturity and ripening assessment, and storage

A commercial orchard comprised of mature open vase trained ‘d’Anjou’ trees [‘d’Anjou’ scion on ‘Bartlett’ seedling rootstock, 6 m × 6 m planting distance, 278 trees/ha with rows in an east-west orientation (Cashmere, WA, USA, 47° 31′ 22.3″ N, 120° 30′ 41.1″ W)] planted in the 1970’s was used for this trial. Trees were chosen for large canopy volume and expected high fruit maturity variability at harvest. Fruit were first sampled from a single tree 2 days prior to harvest to provide an estimate of crop size, defects, and variability of fruit maturity to determine when the best harvest date and amount of fruit in each tree position prior to harvesting the test trees.

To define external and internal canopy zones, a portable spectrometer was used to quantify light intensity in different horizontal layers within tree canopies as previously described [3]. Using this protocol, branches within 15 trees were categorized according to two height levels of approximately 2.0 m and 3.5 m and then midday light measures conducted above each height category. Light measurements were performed in two passes, one on each side of the canopy covering all four quadrants (North-West, North-South, South-West and South-East). The pass length was 6.0 m (3.0 m across the row on both sides North and South of each trunk). During each pass, light-bars were held perpendicular to each row and data were collected at 0.3 m intervals creating a grid of 21 readings × 24 readings (across row × with row). Midday light measures were mainly conducted on the lower level (approximate 2.0 m). Internal fruit were harvested based on measurements taken from the lower level. External fruit were sampled from the outer layer of the canopies on the upper level [3]. Temperature within the canopy was not evaluated.

### Fruit harvest, sorting, and storage

Fruit from each of the two light penetration levels were harvested on September 10, 2014 based on firmness (see below). Fruit from the lowest (internal; < 30% light interception) and highest (external; 70–100% light interception) canopy regions were harvested into separate containers and moved to 4 °C where fruit maturity distribution was assessed on 1013 external and 934 internal fruit. Fruit less than 170 g or greater than 300 g were discarded. Fruit from each tree position were then randomized and stored in controlled atmosphere (CA) (− 0.5 °C, 2 kPa O_2_ and 0.8 kPa CO_2_) for 0, 3, 6, or 8 months beginning on 09/17/2014. Quality and ripeness were assessed, and peel samples taken at 0, 3, 6, and 8 months.

### Tissue sampling

Upon removal from CA, fruit were stored at 0.5 °C, washed with deionized water for 1 min, air dried, and peeled at that temperature within 1 d after fruit were moved into air. At-harvest sorting and sampling was performed on cold fruit. Peel sample replicates were a composite of 15 fruit (5 replicates) per canopy position. Although peel was collected from all portions of the fruit, sunburned portions were avoided. Peel tissue was collected using a sharp potato peeler and immediately frozen in liquid nitrogen. Frozen samples were ground to a fine powder using a rotary mill (A11 basic mill, IKA® Works, Inc. Wilmington, NC, USA) prior to analyses.

### Fruit quality assessment

Sixty fruit from each canopy position were used for fruit quality assessment. At harvest, for each canopy position we estimated/measured the percentage of peel red blush over-color surface, the background color by CIELAB coordinates L* (lightness, 0 = black, 100 = white), a* (green-red), b*(blue-yellow) (Minolta CR-200, Osaka, Japan), from those values Chroma (C), Hue angle (h) were calculated accordingly to McGuire [87] and Nunez–Delicado et al. [88]. Intact fruit ethylene production [89] and superficial scald incidence (visually; % total) were all estimated. Following non-destructive evaluations, firmness (MDT-1 analyzer with an 8 mm diameter probe, Mohr and Associates, Richland, WA), fruit diameter, soluble solid content (refractometer PAL-1, ATAGO, USA Inc., Bellevue, WA, USA), titratable acidity (TIM850 titrator, Radiometer, Lyon, France), and pH were analyzed on the same fruit. Quality of fruit at harvest were not analyzed following 7 days at 23 °C. Quality was also evaluated following 3, 6 and 8 months of CA storage, immediately following CA removal and after 7 days at 23 °C.

### Metabolic profiling

Extraction, instrumental analysis, data extraction, and review were performed as exhaustively detailed in Rudell et al. [11].

Briefly, 3 extractions and 3 instrumental protocols (with protocol consisting of introduction of 2 different volumes) were performed with the goal of approaching a non-targeted, unbiased assessment of freely soluble metabolites regardless of polarity or volatility. Volatile compounds were analyzed from the headspace over 0.5 g of frozen peel powder extracted in 1 mL saturated NaCl solution using a gas chromatograph (Agilent 6890/5975 N, Agilent, Santa Clara, CA, USA) coupled with a single quadrupole mass selective detector and an automated Gerstel multipurpose sampler (MPS; Gerstel, Baltimore, MD, USA) equipped with a dynamic headspace sampler (DHS). For non-polar metabolites, frozen peel powder (0.5 g) was extracted in 80% buffered acetone, partitioned into hexanes, of which the dried extract was dissolved in acetone and analyzed using reversed phase high performance liquid chromatography coupled with a single quadrupole-time of flight mass selective detector (RP-HPLC-QTOF-MS; Agilent 1260 HPLC with 6520 QTOF,) equipped with an atmospheric pressure chemical ionization (APCI) source as described by Leisso et al. [18]. For polar metabolites [11], frozen peel powder (0.1 g) was extracted in buffered methanol containing EDTA and cleaned by partitioning non-polar metabolites into chloroform. The vacuum dried analyte was dissolved in acetonitrile and analyzed using zwitterionic hydrophilic interaction chromatography (ZIC®-pHILIC; EMD Millipore Corporation, Billerica, MA, USA) on the same HPLC-QTOF-MS equipped with an electrospray ionization (ESI) source. Two separate analyses were performed on 1 and 10 μL of each sample to assure quantification was performed within the linear range of the instrument.

### Quality control

A bulk sample of ‘d’Anjou’ peel from a mix of fruit sampled at harvest, stored 8 months in CA, and stored for 8 months in CA and ripened 7 d at 20 °C were extracted and analyzed twice daily for all LC-MS and GC-MS analyses. Mass spectral tags (MSTs) representing both identified and unidentified metabolites were monitored to assure consistent extraction and instrument performance throughout the analysis period. The response of these selected metabolites was required to be less than 10% across the entire experiment. Samples failing to meet this criterion were re-extracted and analyzed.

### Chromatographic mass spectral data extraction and pre-processing

Raw data from each method were processed using MZmine 2.20 [90] to generate a mass spectral tag library for the non-polar and polar data and process and review all data as outlined in Rudell et al. [11]. For polar compounds, the 1 μL analysis was used for all detectable metabolites and the 10 μL analysis for the remaining metabolites not detectable in the 1 μL analysis.

### Statistics and modeling

Differences between canopy positions for all continuous variables were analyzed at each sampling point as well as significance of changes across all storage durations were assessed using the SAS proc. GLM using a Type III sums of squares test (SAS Institute, Cary, NC, USA). Models were considered significantly different if *p* < 0.05. *Post-hoc* means separations were performed using Bonferroni test. Percentages of blushed surface (ranging from 0 to 55%) were first arcsin transformed and analyzed according to Gomez and Gomez [91] prior to using proc. GLM. Binary disorder variables were analyzed using proc. LOGISTIC (SAS Institute, Cary, NC, USA) and then reported as incidence (%) at each storage time point.

Principal components analysis (PCA) was performed on mean centered and standard deviation corrected data using Unscrambler X (Camo, Trondheim, Norway). Undirected, pair-wise, signed correlation networks were generated and overlaid using the Weighted Gene Correlation Network Analysis (WGCNA) package for R [92] using the average among replicates of each variable/timepoint combination. Variable settings for the network model were kept at the default except: softPower = 20 (based on power analysis of 1 to 32) and the adjacency variable and correlation network were calculated using “signed” settings (networkType = “signed” for the soft threshold calculation, type = “signed” for adjacency calculation, and TOMType = “signed” for TOM calculation). No module consolidation was required. Cytoscape 3.4 was used to render the graphical representation of the network generated by WGCNA and overlay module colors. The AllegroLayout 2.2 app was used for the layout design using the “Edge-Repulsive Fruchterman-Reingold” algorithm. The WGCNA edge weight variable was used to supervise the layout algorithm and layout scale and gravity settings were adjusted to render a circular network (“gravity” setting) maximizing module contrast and node density.

## Additional files

Additional file 1: **Table S1.** ‘d’Anjou’ metabolic profile information. Retention time/index, target ion, metabolite identification or classification, identification quality, identification or classification basis, WGCNA network node name, and network module color. (CSV 61 kb)

Additional file 2: **Table S2.** Example spectra of select tentatively identified metabolites. (CSV 3 kb)

## Abbreviations

APCI: Atmospheric pressure chemical ionization
ASG: Acylated steryl glycoside
CTOL: (7*E*,9*E*)-2,6,10-Trimethyl-2,7,9,11-dodecatetraen-6-ol
DGDG: digalactosyldiacylglycerol
EDTA: Ethylenediaminetetraacetic
ESI: Electrospray ionization
GLM: Generalized linear model
MGDG: Monogalactosyldiacylglycerols
PC: Principal component
PCA: Principal components analysis
RP-HPLC-QTOF-MS: Reversed-phase high performance liquid chromatograph-single quadrupole time of flight-mass spectrometer
SG: Steryl glycosides
WGCNA: Weighted Gene Correlation Network Analysis
